# Health-related outcomes of youth sport participation: a systematic review and meta-analysis

**DOI:** 10.1186/s12966-025-01792-x

**Published:** 2025-07-01

**Authors:** Dennis Bengtsson, Joar Svensson, Virginia Wiman, Andreas Stenling, Erik Lundkvist, Andreas Ivarsson

**Affiliations:** 1 School of Health and Welfare, Halmstad University, Kristian IV:S Väg 3, 301 18 Halmstad, Sweden; 2 Department of Psychology, Umeå University, Sweden, 901 87 Umeå, Sweden; 3 Department of Sports Science and Physical Education, University of Agder, Agder, Norway; 4 Umeå School of Sport Sciences, Umeå University, Sweden, 901 87 Umeå, Sweden

**Keywords:** Youth sport, Effect, Longitudinal, Health

## Abstract

**Background:**

Participating in youth sports can benefit individuals’ psychological (e.g., fewer depressive symptoms, improved self-esteem), social (e.g., improved social skills, learning to work with others as a team), and physical health-related outcomes (e.g., higher physical activity levels, lower body fat), aligning with global sustainable development goals. Nevertheless, little is known about the magnitude concerning the effects of youth sport participation on such health-related outcomes compared with nonparticipation over time from childhood to adulthood. In this paper, we systematically review the extant longitudinal research and estimate the effects of youth sport participation on several psychological, physical, and social outcomes compared with nonparticipation.

**Methods:**

Electronic database searches were employed to identify English-language peer-reviewed studies published from the earliest date until October 4, 2024. By using a priori criteria for inclusion and exclusion, we included 46 out of 4588 identified individual studies in the systematic review and 38 of the eligible studies for calculation of Cohen’s *d* effect size estimates.

**Results:**

Together, the follow-up measurements of the included studies varied from 1 to 54 years after baseline, and the sample sizes ranged from 76 to over 50,000 participants. The meta-analysis revealed that youth sport participation had positive and statistically significant low- to medium-sized effects on physical activity, health and wellbeing, and negative small- to medium-sized effects on unhealthy body composition and mental ill-being over time.

**Conclusions:**

This study provides evidence that participating in youth sports can have health-promoting effects throughout childhood, adolescence, and adulthood. This advocates for collaborative efforts among national governments, sport governing bodies, communities, and sports clubs to create an accessible and inclusive youth sport environment where young people can thrive and reap the health benefits of sport participation.

**Supplementary Information:**

The online version contains supplementary material available at 10.1186/s12966-025-01792-x.

## Background

Promoting children’s and adolescents’ health and well-being through regular physical activity is a global priority that aligns with the World Health Organizations (WHO) sustainable development goals [1]. Youth is a critical period for establishing healthy behaviors, including being physically active through youth sport participation [2]. In the extant literature, the argument for positive short- and long-term health outcomes of sport participation mainly pertains to physical activity behaviors and not youth sports per se (cf. [3]). To better understand the potential benefits of youth sport participation Eime et al. [4] conducted a systematic review focusing on the psychological and social benefits, reporting that the sporting environment itself can foster various positive developmental and health-related outcomes, such as improved mental health (e.g., fewer depressive symptoms and better self-esteem) and social wellbeing (e.g., teamwork and social competence). However, Eime et al. [4] noted a lack of studies with longitudinal designs and control groups, calling for research to address these shortcomings.

In recent years, researchers have examined the outcomes of youth sport participation, finding associations with lower body fat and better fitness (VO2 max and max sit ups in 30 s) compared with nonparticipants [5] and higher levels of future habitual physical activity [6]. Furthermore, meta-analyses have shown significant effects on blood pressure [7]; decreased rates of cigarette, tobacco, and alcohol use (e.g., [8, 9]); and the potential of youth sport participation as a protective mechanism against anxiety and depressive symptoms among adolescents [10]. However, these findings primarily stem from a mix of cross-sectional and retrospective studies, with relatively few longitudinal studies, limiting the ability to draw valid inferences about temporal relationships [11]. Although causal claims are rarely made explicitly, prior observational studies and meta-analyses (e.g., [5–10]) have investigated the potential effects of sport participation (i.e., the exposure) on health-related outcomes, and with comparison groups (e.g., [5, 9, 10]), thereby implicitly invoking causal mechanisms despite methodological challenges in establishing temporal precedence [12]. By focusing on longitudinal research and comparing youth sport participants with nonparticipants, this study can more efficiently conduct a meta-analysis of the predictive associations between youth sport participation on future health-related outcomes [12, 13]. Such investigations are needed to advance understanding of the potential health benefits that can be reaped into adulthood. Hence, the aim of this systematic review and meta-analysis was to investigate the longitudinal effects of youth sport participation on psychological, physical, and social outcomes compared with nonparticipation.

## Methods

This study was conducted in accordance with the Preferred Reporting Items for Systematic Reviews and Meta-Analyses (PRISMA) guidelines [14].

### Literature search strategy

**Fig. 1:**
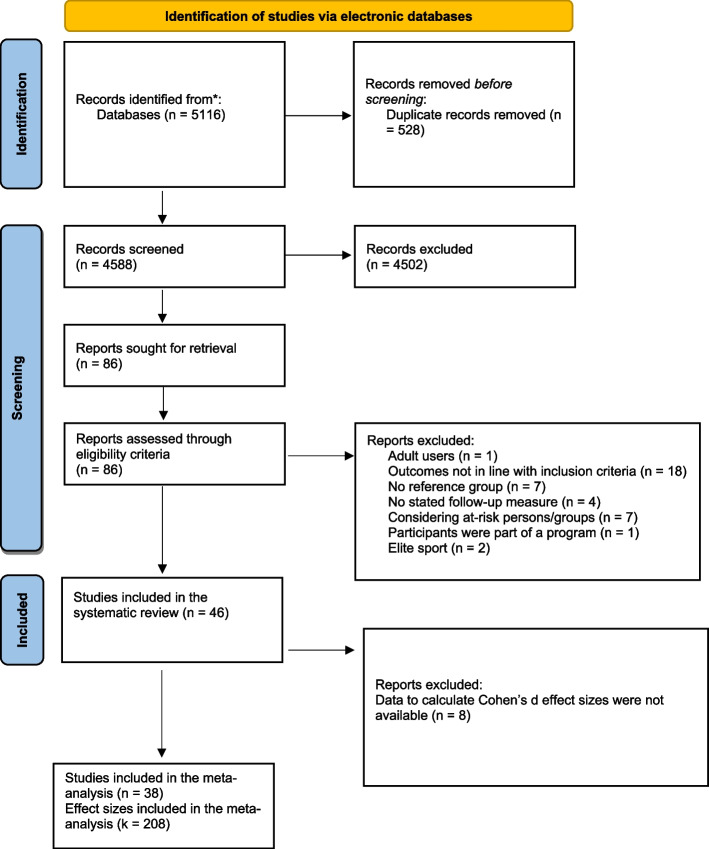
PRISMA Flow Diagram

A summary of the search process is illustrated in Fig. 1. As we aimed to capture all relevant longitudinal studies on youth sport participation and health-related outcomes, we performed electronic searches of full-text and peer-reviewed articles from the earliest reported date until the 4th of October 2024 in the following databases: the PsychINFO, PubMed, Scopus, and EBSCOhost online databases (CINAHL, Eric, Medline, and SportDiscuss). The search string was informed by previous studies (e.g., [4]) and was developed in consultation with a university librarian. Overall, the search strategy consisted of a combination of five separate groupings of the included terms (Group 1: sport*), considering the players (Group 2: youth* OR adolescence* OR teen*), and the research design (Group 3: longitudinal OR prospective). The outcomes searched were both general (Group 4: consequence OR value* or benefit* OR effect* or outcome*) and specific (Group 5: psychology OR physiology OR “biomechanical function” OR depress* OR stress* OR anxiety* OR happiness OR emotion* OR “quality of life” OR “social health” OR “social resources*” OR well* OR “social connect*” OR “social function*” OR “life satisfaction” OR “mental health” OR “physiological health” OR sociology OR social* OR “prosocial behavior” OR “antisocial behavior” OR lifestyle OR “health behavior*”). In the final search, each of the groupings was combined with the operator “AND” in the electronic databases. The search terms were arranged as relevant MeSH terms or subject headings where suitable in the electronic database searches.

### Criteria and screening process

The Rayyan web application was used to manage records retrieved from the literature search (https://rayyan.qrci.org). Initially, studies were included if they (1) were original articles published in peer-reviewed journals; (2) were written in the English language; (3) addressed psychological, social, or physical consequences (i.e., positive or negative) from organized youth sport participation; and (4) stated that they specifically investigated children and/or adolescents from baseline (i.e., up to 19 years; [15]). Like previous research in the field [4], we defined sport participation as engagement in organized and competitive activities, generally accepted as sports, either individually or in a team, aimed at achieving a result through physical exertion and/or skill. The exclusion criteria were as follows: (1) studies that did not investigate sport participation per se (e.g., physical education, exercise, recreation); (2) studies that focused on elite sports participants (e.g., junior or senior level); (3) studies that did not include any reference group for comparison (i.e., no sport participation); (4) studies that included at-risk populations (e.g., drug users); (5) studies that addressed adult users (e.g., coaches, sport administrators, or spectators); (6) studies about sport development programs with an educational objective; and (7) studies with no follow-up (i.e., longitudinal or prospective) measurements. The electronic database search and assessment of the retrieved titles and abstracts of the records were independently performed by one author (masked for review). In the full-text screening stage, five authors independently screened each article according to the eligibility criteria. Any disagreements were resolved through consensus (see Fig. 1 for an overview of the screening process).

### Data extraction

The articles were alphabetically sorted in an Excel sheet and assigned a bibliographical code to differentiate the articles included in the review and other references. Following the recommendations of Taylor et al. [16], the first author extracted the data into an extraction form which was then cross-checked by the second author. The specific information extracted from each study was the author(s), year of publication, aim, design, method and follow-up measurements, sample characteristics, cohort, study aim, type of sport participation, other physical activity variables, theoretical construct, and key findings and outcomes in relation to the psychological, social, and/or physical factors (see Additional file 1). After the data extraction, four authors (masked for review) reviewed, discussed, and later grouped the individual study outcome variables into 10 outcome categories for meta-analysis (see Table 1). To estimate the effect size of our outcome categories, each category required effect sizes from at least two eligible studies that were combined if they were regarded as being ‘sufficiently similar’ [17]. Supplementary information was requested from corresponding authors when the data necessary for the meta-analyses were insufficiently described in an article. Three out of eleven authors provided us with additional data upon request.

**Table 1:** Distribution of Individual Studies Across Meta-Analyzed Variables

Physical Outcomes
Anthropometric measurements: Body mass index (BMI; 5 studies), Body mass index Z-score (BMIz; 1 study), Waist circumference (2 studies), Weight (2 studies) Biomedical risk factors: Bone mineral content (1 study), Diastolic blood pressure (1 study), EGIR metabolic syndrome (Abdominal obesity, high blood pressure, high triglycerides, low HDL cholesterol, and high fasting glucose levels; 1 study), Systolic blood pressure (1 study) Unhealthy body composition: Body fat (2 studies), Fat mass (1 study), Fat mass index (FMI; 1 study), Lean body mass (2 studies), Lean mass index (1 study), Skinfold thickness (1 study) Injuries: Fractures (1 study), Injury hospitalization (1 study) Lifestyle risk factors: Alcohol consumption (3 studies), Fruit and vegetables (1 study), Healthy habits (smoking, alcohol consumption, diet, and physical activity [changed direction]; 1 study), High fat diet (1 study), High sugar drinks (1 study), Smoking (1 study), Screen time (1 study), Sedentary behaviors (1 study) Physical activity: Moderate to vigorous physical activities (MVPA; 4 studies), Physical activity (PA; 9 studies), Physical inactivity in adulthood (1 study), Vigorous-intensity physical activity (VPA; 1 study) Physical Fitness: Time to exhaustion (1 study), VO2 peak (1 study) VO2 (1 study)
Psychological Outcomes
Health and wellbeing: Autonomy (1 study), Mental health (3 studies), Parent-reported health-related quality of life (PedsQL; 1 study), Physical well-being (1 study), Psychological well-being (1 study), Self-esteem (1 study), Self-perceptions (1 study), Physical Health (1 study) Mental ill-being: Agoraphobia (1 study), Anxiety (3 studies), Chronic High Job Strain (1 study), Depressive symptoms (8 studies), Internalizing problems (2 studies), Panic disorder (2 studies), Social anxiety (2 studies), Social phobia (1 study), Stress (3 studies)
Social outcomes
Social problems: Externalizing problems (3 studies), Loneliness (1 study), Prosocial behavior (changed direction; 1 study), Reactivity (1 study)

*Note.* Individual studies may contribute data to multiple meta-analyzed variables

### Risk of bias appraisal of included studies

Following the guidelines of previous research [18, 19], the Risk of Bias Assessment Tool for Nonrandomized Studies (RoBANS) was used to evaluate the risk of bias in each of the included studies. The risk of bias assessment was conducted by one primary author independently and in consultation with a second author (see Additional file 3).

### Meta-analysis

Studies from the systematic review that reported sufficient data to calculate Cohen’s *d* effect sizes were added to the meta-analysis. The estimated effect sizes were computed by using mean values, odds ratios, correlation coefficients, standard deviations, and sample sizes (see Table 2). As the included studies varied in their number of effect sizes, groups, and measurements of relevance for the current study, dependencies were created, and cluster effects were added to estimate the overall mean effect size for each meta-analyzed outcome, as has been previously recommended [20]. Consequently, we used three-level meta-analytical random effects models with cluster robust variance estimation, including restricted maximum likelihood estimation (e.g., [21, 22]). We interpreted the effect sizes according to the suggestions of Lovakov and Agadullina [23]: 0.15 < small, 0.36 < medium, and 0.56 < large. Egger’s regression test and visual inspection of funnel plots were performed to investigate potential publication bias and asymmetry in the effect size distribution from the meta-analysis (see Additional file 4). A significance level of α = 0.05 and 95% confidence intervals (CIs) were used for all analyses. We conducted the meta-analysis via the R packages *metaphor* and *clubSandwich* [24].

## Results

### Study characteristics

A total of 5116 records were found through the literature search, and 528 duplicate records were removed, resulting in 4588 records for screening. Subsequently, 4502 records were removed through the initial title and abstract screening. The full texts of the remaining 86 studies were evaluated, resulting in 46 studies that met the eligibility criteria and were included in the narrative synthesis. A summary of the process of study selection is illustrated in Fig. 1.

All the included studies were quantitative and longitudinal. The time between data collection points varied from 1 to 54 years between baseline and the last follow-up measurement. The sample sizes varied from 76 to over 50,000 youth sport participants. The studies were conducted in Western countries (e.g., Australia, Denmark, USA), and the age ranges at baseline (3–19) and last follow-up (7–65) varied considerably. The different types of sports ranged from individual karate [25], gymnastics [26], and swimming [27] to team sports such as ice hockey [26], baseball [27], and football [25]. The comparison groups consisted of nonsport participants. An overview of all the studies included is provided in our Additional file 1.

### Meta-analysis

The meta-analysis included 38 of 46 (83%) studies from the systematic review. The meta-analysis revealed significant effects of youth sport participation on physical, psychological, and social outcomes (see Table 2). Compared to nonparticipants, youth sport participants reported higher physical activity levels, better health and well-being, and fewer symptoms of mental ill-being and unhealthy body composition. We found no statistically significant effects on biomedical risk factors, physical fitness, anthropometric measurements, lifestyle risk factors, injuries, or social problems.

**Table 2:** Meta-analysis on the Outcomes of Youth Sport Participation Compared to Non-participation

Outcome	n(*k*)	*d*	SE	95% CI	*p*
**Physical outcomes**					
Anthropometric measurements	7(*17*)	−0.06	.04	−0.18, 0.06	.233
Biomedical risk factors	3(*10*)	0.28	.18	−0.49, 1.05	.260
Unhealthy body composition	5(*15*)	−0.21	.04	−0.33, −0.09	.009
Injuries	2(*5*)	−0.26	.13	−1.86, 1.35	.289
Lifestyle risk factors	5(*27*)	−0.05	.06	−0.22, 0.12	.472
Physical activity	14(*40*)	0.24	.05	0.13, 0.34	<.001
Physical fitness	2(*7*)	0.44	.05	−0.20, 1.07	.072
**Psychological outcomes**					
Mental ill-being	12(*44*)	−0.25	.11	−0.49, −0.02	.039
Health and Wellbeing	5(*35*)	0.23	.05	0.10, 0.35	.008
**Social outcomes**					
Social problems	5(*8*)	−0.08	.05	−0.23, 0.06	.167

*Note. k* indicates the number of individual effects and *n* the number of individual studies in each meta-analyzed outcome

### Physical outcomes

Our meta-analysis revealed a statistically significant small to medium negative effect of youth sport participation on unhealthy body composition (*d* = −0.21, 95% CI [−0.33, −0.09], *p* = 0.009), including lower levels of adiposity (e.g., [28, 29]*) and higher levels of lean mass (e.g., [20, 30]). The results also revealed a statistically significant small to medium positive effect on physical activity (*d* = 0.24, 95% CI [0.13, 0.34], *p* < 0.001), with studies investigating both overall physical activity (e.g., [28, 31, 32]*) and moderate-to-vigorous physical activity (e.g., [30, 33, 34]*). The analyses revealed no statistically significant effects on anthropometric measurements, biomedical risk factors, injuries, lifestyle risk factors, physical activity, or physical fitness. Seven studies focused on anthropometric measurements such as BMI (e.g., [35, 36]*), waist circumference [28, 37]*, and weight [30]*. Three studies focused on biomedical risk factors such as bone mass density [31]*, metabolic syndrome [38]*, and blood pressure [28]. Two studies focused on injuries and investigated hospitalization [39]* and fractures [40]*. Five studies focused on lifestyle factors, including alcohol use and smoking [31, 41]*, sedentary behaviors [26], diet and screen time [43]*. Finally, physical fitness was the focus of two studies investigating time to exhaustion [44]* and peak Vo2 [45]*.

### Psychological and social outcomes

Overall, our analyses revealed a statistically significant positive small to medium effect of youth sport participation on health and well-being (*d* = 0.23, 95% CI [0.10, 0.35], *p* = 0.008) and a small to medium negative effect on mental ill-being (*d* = −0.25, 95% CI [−0.49, −0.02], *p* = 0.039). Five studies focused on health and well-being outcomes, such as mental health [30]*, [40]*, [46]*, health-related quality of life [30]*, [47]*, and self-esteem [44]*. Symptoms of mental ill-being were the focus of 12 studies investigating outcomes such as anxiety symptoms (e.g., [48]*, [49]*), depressive symptoms (e.g., [50]*, [51]*), and stress symptoms (e.g., [26], [30]*). With respect to social outcomes, our analyses revealed no statistically significant effect of youth sport participation. In total, five studies focused on social outcomes investigating reactivity [25], loneliness [50]*, externalizing problems [27], [52]*, [53]*, and prosocial behavior [53]*.

### Publication bias

Egger’s regression test was not statistically significant, indicating that there was no major risk of publication bias (*β*_0=_ 0.91, *SE* = 0.50, *p* = 0.07), and the inspected funnel plot revealed that most of the estimated effect sizes were symmetrically gathered around the overall effect within the 95% confidence interval.

## Discussion

This study aimed to systematically review and estimate the longitudinal effects of youth sport participation on psychological, physical, and social outcomes compared with nonparticipation, filling a critical research gap highlighted in previous research [4]. We identified 48 prospective studies with follow-up times ranging from 9 months to 54 years. Previous research has revealed statistically significant effects of youth sport participation on psychological outcomes, including fewer symptoms of anxiety and depression [10], positive effects on social outcomes (e.g., social functioning; [4]), and lower body fat compared with non-sport participants [5]. Our meta-analysis extends the current evidence regarding the effects of youth sport participation on players’ physical activity levels, body composition, health and wellbeing, and mental ill-being over time compared with nonparticipants.

There are several potential explanations for why organized youth sports may protect players from ill health and facilitate positive health-related outcomes, making them important for the prosperity of the young population [1, 2]. Based on our findings, the potential mechanisms can be divided into intrapersonal, interpersonal, and environmental factors [4]. At the intrapersonal level, sport participation can expose youth players to physiological sensations similar to those experienced during anxiety (e.g., hyperventilation; [48]*). Over time, this exposure may desensitize youth players to these sensations and perceive them as more tolerable and harmless [48]*. Sport participation can also facilitate the development of resilience [54] and life skills, helping individuals cope with their challenges [55]. Lastly, in addition to being a source of exercise and helping individuals increase their physical activity [56]*, sport participation may help young individuals develop more muscle mass and reduce fat mass (e.g., [35]*, [42]*). It is important to note that intrapersonal factors may also moderate the relationship between sport participation and health outcomes. For example, Panza et al. [10] found that the negative association between sport participation and anxiety was stronger in studies with a higher proportion of male participants, whereas the negative association with depressive symptoms was more pronounced in samples of older adolescents. This highlights the relevance for future research to include developmentally relevant demographic moderators in longitudinal research on mental health-related outcomes of youth sport participation.

At the interpersonal level, youth sport participation may foster social competence and peer connections, which can help protect against the development of depressive symptoms [51]*, [57]* and support individuals sustained mental health [49]. This can be promoted through positive youth sport environments wherein coaches provide players with opportunities to develop competency and nurture their needs to achieve a sense of sport mastery, intrinsic motivation, and self-esteem, which can facilitate team social cohesion [46]*, [58]. Systematic reviews and meta-analyses indicate that these mental health-related benefits are more pronounced in team sports than individual sports [4, 9, 59] and this advantage appears to extend into adulthood regardless of competitive level [60]. This highlights the potential of sport environments, particularly those with an inherent social nature, to supply players with supportive social networks [4, 61, 62], which in turn can serve as long-term health-promoting resources as long as individuals remain engaged [9, 10, 62]. This also highlights the relevance of intervention studies that facilitate nurturing climates in youth sport settings focused on factors that impact sustained youth sport participation (e.g., intrinsic motivation, coach support, peer support, parental support; [63, 64]). For example, it seems that intrinsically motivated players who have their needs for competence, autonomy, and relatedness satisfied—and experience support from their coach—are more likely to continue their sport participation [64]. Based on the tenets of self-determination theory [65], coaches who are trained to adopt a need-supportive style can increase youth sport participants'intrinsic motivation and sport engagement [66], suggesting one promising theory-based intervention strategy focused on facilitating nurturing interpersonal coach-player relations [58].

Our meta-analysis confirmed that youth sport participation is associated with higher levels of future physical activity [3]. This suggests that being a youth sport participant is inherently linked to physical activity behaviors later in life [2], potentially explaining their higher activity levels than those of nonparticipants. These findings also highlight that health behaviors, such as physical activity, which are facilitated during childhood and adolescence, may continue into adulthood as a healthy habit [67]*. In contrast with previous meta-analyses (e.g., [7–9]), we found no statistically significant associations between youth sport participation and lifestyle risk factors (e.g., smoking behaviors and alcohol consumption) or biomedical risk factors (e.g., metabolic syndrome and high blood pressure). This may be explained by the methodological differences between our study and previous studies. For example, in contrast to previous meta-analyses (e.g., [7–9]), we explicitly estimated the longitudinal effects of youth sport participation on physical outcomes compared with nonsport participants. Furthermore, there are potentially more potent factors than sport participation that can explain variance in the outcomes. For example, biomedical risk factors such as blood pressure are impacted by genetic factors [68], whereas other factors such as diet [69] and alcohol use are largely impacted by expectancies [70]. As such, the effects of other variables may have a greater impact on some of the health-related outcomes than youth sport participation does, especially over time. Conceptually, the findings of this study and previous research [4, 9, 10, 58, 60–64] underscores the relevance of investigating factors that predict continued youth sport participation and, in turn, whether these collectively predict long-term physical, psychological, and social health-related outcomes.

### Limitations

This systematic review and meta-analysis has several limitations that need to be considered. First, the limit to articles reporting only in the English language may have contributed to the potential loss of eligible studies written in other languages. As with any review of the literature within a field, there is also uncertainty as to how well the search strategy has identified all studies in relation to the aim of this study. However, the literature search was conducted in both comprehensive and topic-related databases, using search strings inspired by Eime et al. [4] in consultation with a university librarian.

Another concern regards the many studies that were classified, according to the RoBANS guidelines, as having a high risk of performance bias owing to the use of self-reported measurements of exposure (e.g., [18]). However, psychological and social health-related outcomes (e.g., perceived depressive symptoms, anxiety, social competence) may be challenging to measure in other and more objective ways in nonexperimental research (e.g., compared with physical activity levels).

Moreover, a meta-analysis is only as robust as the studies it is based on, and its results should be interpreted with consideration of its limitations. Several of our meta-analyzed outcomes were based on a relatively small number of studies, posing a limitation compared with a more robust analysis including larger numbers of studies and effect sizes [22]. This also restricted our capacity to explore heterogeneity and conduct moderator analyses (e.g., type of sport, duration of participation, sex, age), representing a gap for future research. We were also unable to account for dynamic patterns in sport participation, such as the timing of sport initiation or dropout (e.g., due to mental ill-being), or whether participation mediates other associations over time.

Those who continue their sport participation may differ systematically from nonparticipants in unobserved ways, and it is important to emphasize that this study does not meet all criteria for causal inference. Without explicit causal modeling or experimental designs, longitudinal data may be affected by unmeasured confounders (e.g., sex, parental socioeconomic status) or selection bias (e.g., healthier or more motivated youth remaining in sport) related to the exposure (i.e., sport participation vs. nonparticipation), which in turn may influence the outcomes [10–12, 71]. Thus, the reliance on observational data in our meta-analysis introduces a risk of overstating causal claims [11], and interpretations of sport participation as promoting long-term health outcomes should be treated with caution. To advance causal understanding in the field, future primary studies should consider quasi-experimental designs and statistical approaches (e.g., natural experiments, instrumental variable techniques, or propensity score methods) that can strengthen causal inferences concerning the effects of youth sport participation on health-related outcomes [11].

Future meta-analyses of observational studies could also adopt instrumental variable (IV)-based meta-analytic structural equation modeling (MASEM) to strengthen causal inference. This approach allows estimation of the effect of sport participation (predictor, X) on health-related outcomes (e.g., adult physical activity levels, Y) while addressing typical types of endogeneity (e.g., omitted variable bias) using valid IVs—variables having a direct effect on X but affecting Y only indirectly through X. Implementing IV-based MASEM requires identifying valid and consistently reported variables across primary studies, which may necessitate a more selective pool of studies, and a narrower set of variables focused on key predictors and outcomes [72].

### Practical Implications

Youth sport participation seems to have a positive effect on several health-related outcomes, some of which may persist after sport discontinuation. It is therefore important to engage as many children and adolescents as possible in organized sports so that they may reap potential long-term benefits. However, barriers to participation—such as those related to socioeconomic status, age, and gender—can contribute to sport discontinuation [71, 73, 74]. Therefore, stakeholders, including sport organizations, governing bodies, municipalities, and governments, should collaborate to expand opportunities for young people to engage in organized sport over time. For example, investing in accessible community-based sport programs can provide children and adolescents with meaningful opportunities to be physically active, adopt healthier lifestyle habits, develop social skills, and reduce engagement in risk behaviors such as substance use [75]. Stakeholders should prioritize the development and sustained support of such programs to ensure the benefits of sport participation are accessible to all youth, regardless of demographic background. From a research perspective, evaluations of these initiatives could adopt natural experiment designs to compare health-related outcomes before and after program exposure. Three of our proposed explanations for the benefits associated with youth sport participation are improved body composition through increased exercise (leading to increased physical activity, muscle mass, and lower body fat; [26, 35, 56]*, well-being through opportunities to improve and develop competency (leading to improved self-esteem; [46]*), and decreased ill-being through inoculation to symptoms of anxiety and through opportunities to build social connections and improve social competencies [48, 49, 51, 57]*. These mechanisms are not necessarily limited to organized sports. For example, the benefits of increased exercise can be attained through resistance training and cardiovascular training (e.g., [76, 77]), which, in turn, can promote subjective well-being (e.g., [78]). Hence, similar benefits may also be promoted through other accessible forms of physical activity, such as physical education, school-based movement breaks, or non-competitive community-based programs—especially for youth not engaged in organized sports [2, 76–78].

## Conclusions

The results from our systematic review and meta-analysis indicate that individuals who participate in youth sports throughout childhood, adolescence, and adulthood engage in more physical activity, report better health and well-being, have a healthier body composition, and report less mental ill-being over time compared to nonparticipants. In other words, continuous engagement help individuals meet recommended physical activity levels and gain its associated benefits (e.g., reduced body fat, improved physical health, increased physical activity levels) while providing an arena that can buffer against psychosocial ill-health (e.g., depressive symptoms, stress, anxiety). This highlights the importance of youth sport environments that support players’ health-related needs to sustain their sport participation (e.g., supportive social networks, intrinsic motivation; [58, 63, 64]). Stakeholders such as sport clubs, sport federation bodies, and national governments could collaborate to promote initiatives that nurture such positive youth sport environments and launch programs that provide children and adolescents opportunities to reap the long-term benefits of sport participation across the lifespan.

## Supplementary Information

Supplementary Material 1.

Supplementary Material 2.

Supplementary Material 3.

Supplementary Material 4.

## Data Availability

This study was not preregistered, and the materials, methods, and data used are available upon request from the corresponding authors.

## Abbreviations

BMI: Body Mass Index
CI: Confidence Interval
IV: Instrumental Variables
MASEM: Meta-analytic Structural Equation Modeling
PRISMA: Preferred Reporting Items for Systematic Reviews and Meta-analyses statement, RoBANS—Risk of Bias, Assessment Tool for Nonrandomized Studies
VO2: Maximal Oxygen Consumption
WHO: World Health Organization
