# Supplementary material for: Health-related outcomes of youth sport participation: a systematic review and meta-analysis
Source: Int J Behav Nutr Phys Act. 2025 Jul 1;22:89. doi: 10.1186/s12966-025-01792-x (PMC12220085; doi:10.1186/s12966-025-01792-x)
Supplement: Supplementary file 1 — Supplementary Material 1. [file 12966_2025_1792_MOESM1_ESM.docx]

**Additional file 1**

*Key Characteristics of Included Studies*

| **Ref. & Year** | **Method/follow-up (measure)** | **Participants (N, age, sport, country)** | **Aim** | **Cohort, comparison** | **Key findings** | **Outcomes** |
| --- | --- | --- | --- | --- | --- | --- |
| *Allen et al. (2015) | Long. baseline-measure, follow-up after 24 months, 2 years total | 7818,  Australia, 6 yrs and 10 yrs, organized sport | Explore the potential contribution of an active childhood to personality trait stability and change in two cohorts of children | Child., parent, no sport | Sport participation in children facilitate desirable personality traits (i.e. decrease in introversion & less increase in reactivity) during childhood | Internalizing problems |
| *Ashdown-Franks et al. (2017) | Prospective cohort study. 20 survey cycles during high school (grade 7-11) every 3^rd^ month, 8 years total | 781, Canada, 12-13 yrs (start), 20 yrs (end), organized and non-organized sport | Examine the longitudinal associations between sport participation during high-school and symptoms of panic disorder, GAD, social phobia and agoraphobia in young adulthood | Adol., no sport | Consistent involvement in sport during high school is beneficial for reduced levels of anxiety-related symptoms | Anxiety |
| *Basterfield et al. (2015) | Long. & cross-sectional. 3 measures (baseline, middle, end), 6 years total | 609, United Kingdom,  7 yrs (start), 12 yrs (end), organized sport | Investigate longitudinal associations between sports club participation, physical activity, and adiposity | Child., no sport | Sports club participation in adolescence may be associated with decreased levels of adiposity and increased levels of physical activity over time | Physical activity  Sedentary behaviour  Adiposity |
| Bedard et al. (2020) | Long. 8 waves / measures every spring and fall, 4 years total | 2278, Canada, 10 yrs (start), 13-14 yrs (end), organized sport | Evaluate the association between sport participation and self-perceived social competence | Child., Adol., no sport | Higher participation in sport is associated with gains in perceived social competence during late childhood to early adolescence. | Social competence |
| *Brière et al. (2018) | Long. 2 measures (baseline – end), 1 year total | 17550, Canada, Grade 7-10 (start), organized sport | Determine whether sport participation independently predicts lower depressive symptoms, social anxiety symptoms and loneliness | Adol., no sport | Sport participation predicts small reductions in adolescent psychological distress and difficulties. | Depressive symptoms  Social anxiety symptoms  Loneliness |
| *Brunborg et al. (2022) | Long. 4 waves / measures, 4 years total | 3425, Norway, 12-15 yrs (start), 15-18 yrs (end), organized sport | Examine if sport participation is a risk factor for alcohol use among adolescents. | Adol., no sport | Increased sport participation was weakly associated with change in alcohol use suggesting lower consumption over time. | Alcohol use |
| *Brunet et al. (2013) | Long. 20 survey cycles during secondary school every 3^rd^ month on average (5 years), with an additional follow-up after 2 years (7 years total) | 860, Canada, 12-13 yrs (start), 18-24 yrs (end), organized team-sport | Assess the longitudinal associations of MVPA and team sports during secondary school with depressive symptoms in young adulthood | Adol., no sport | Physical activity within sport contexts should be encouraged so that young adults may experience fewer depressive symptoms | Depressive symptoms |
| *Deshpande et al. (2017) | Long. 2 recorded measures for this study (54 years total) | 3904, USA, Late adol (start) – 65 yrs (end), organized team-sport | Estimate the association of playing high school football with cognitive impairment and depression at 65 years of age | Adol., non-collision sport or no sport | No associations between playing football in high school and increased cognitive impairment or depression in life on average | Depression  Cognitive impairment  Anxiety  Alcohol use |
| Dodge & Lambert(2009) | Long. 3 waves/ measures (7 years total) | 8152, USA, grade 7^th^-12^th^ (start), 6 yrs later (end), organized sport | Examine the relationship between sport participation during adolescence and physical activity and subjective health in young adulthood | Adol., no sport, exercise or, Biking/  skating or physical activity | Participation in an organized sport during adolescence was associated with higher levels of physical activity and better subjective health during young adulthood | Physical activity  General health. |
| Doré et al. (2019) | Long. measure every 4 months (sport participation), mental health had 1 measure at end (6 years total) | 318, Canada, 10-11 yrs (start), 15-16 yrs (end), organized and non-organized sport | Investigate the longitudinal association between number of years with a recreational or performance sport profile and mental health during adolescence | Child., no sport | Both recreational and performance sport in childhood/early adolescence were positively associated with mental health in late adolescence | Mental health |
| Fay & Lerner, (2013) | Long. 3 waves/ measures (for each grade; 3 years total) | 1050, USA, 9th grade (start), 11^th^ grade (end), organized sport | Contribute to the eating disorder and adolescent development literature both theoretically and methodologically | Adol., no sport | Sport participation was negatively related to depressive symptoms | Depressive symptoms |
| *Gallant et al. (2022) | Long. measures from 23 cycles (18 years total) | 655, Canada, 7^th^ grade (start), average 31 yrs (end), single and multi-sport | Investigate natural trajectories of sport participation in adolescence and its associations with physical activity and health outcomes in young adults | Adol., no sport | Trajectories of sport participation throughout adolescence is associated with higher levels of self-reported physical activity in young adulthood. | Physical activity  Physical fitness |
| Golle et al. (2014) | Long. measures every year for 4 weeks each time (i.e., March – April; 4 years total) | 172, Germany, 9-12 yrs (start), 13-16 yrs (end), organized sport | Examine the effects of living area and sports club participation on physical fitness development in primary school children from grade 3 to 6 | Child., no sport | Sport club programs with appealing arrangements appear to promote physical fitness in children living in rural areas | Physical fitness |
| *Hardie Murphy et al. (2017) | Long. 2 measures (T1, T2; 5 years total) | 873, Republic of Ireland, 10-18 yrs (start),15-23 yrs (end), organized sport | Evaluate domains of PA in youth as predictors of PA 5 years later (study 1). Study 2 aimed to (1) assess uptake, dropout and participation levels in youth sports; and (2) determine reasons for change in sports participation. | Child., adol., no sport | Participation in youth sport club or extracurricular youth sport significantly predicted physical activity five years later | Physical activity |
| *Haynes et al. (2021) | Long. 5 measures/ time points (ages: 5, 8, 10, 14, 17, 28; 13 years total) | 402, Australia, 5-6 yrs (start), 28-29 yrs (end), organized sport | Investigate whether sport participation during the developmental stages of life is associated with adult cardiorespiratory fitness | Adol., no sport | In males, sport participation (consistent & joiners) had greater fitness than dropouts.  In females, sport participation had greater fitness than non-participants. | Physical fitness |
| *Hebert et al. (2015) | Prospective cohort. Measured physical activity in 7 days/2 occasions. Parents reported child sport-participation (1 year total) | 1124, Denmark, 1^st^-6^th^ grade (one-year time interval), organized sport | Explore the associations of organized leisure-time sport participation with overall physical activity levels and health related physical activity guideline concordance | Child., parents, no sport | Organized leisure-time sports participation may increase overall health-related physical activity levels | Physical activity |
| *Howie et al. (2016) | Long. 6 measures (ages: 8, 10, 14, 17, 20, 22; 14 years total) | 1679, Australia, 5 yrs (start), 20 yrs (end), organized sport | Identify unique organized sport trajectories from early childhood to late adolescence in an Australian pregnancy cohort, the Raine Study | Child., parents, no sport | Differences in health outcomes between trajectory classes, such as participants with consistent sport participation had desirable health outcomes in young adulthood | Physical activity  Adiposity  Physical health  Mental health  Depression  Anxiety  Stress |
| *Jewett et al. (2014) | Long. 20 survey cycles during secondary school every 3^rd^ month on average (5 years). Cycle 21 (follow-up) measured 3 years after cycle 20, (10 years total) | 853, Canada, 7^th^ grade (start) young adulthood (end), organized sport | Examine the association between participation in school sport during adolescence and mental health in early adulthood | Adol., no sport | School sport participation may protect against poor mental health in early adult-  hood | Mental health  Depressive symptoms  Stress |
| *Kjønniksen et al. (2009) | Long. 8 measures (ages: 13-16, 18, 19, 21, 23; 10 years total) | 630, Norway, 13 yrs (start), 23 yrs (end), organized sport | Examine whether early and sustained organized youth sport during childhood and adolescence predicts the frequency of leisure-time physical activity (PA) at age 23 years | Adol., no sport | Organized youth sports  during childhood and adolescence was positively related to higher frequency of leisure-time PA in young adulthood | Physical activity |
| *Lagestad & Mehus (2018) | Long. measures carried out during a 2 month period annually (participants divided in 3 subgroups; 5 years total) | 76, Norway, 14 yrs (start), 19 yrs (end), organized and non-organized sport | Examine how regular participation in organized and unorganized PA affect the development of adolescents’ CRF (peak oxygen consumption [VO2peak]), when controlled for interaction effects of sex | Adol., no sport | Adolescents participating in  organized sport had higher VO2-peak values than adolescents participating in unorganized PA and those with no weekly PA over time | Physical fitness |
| *Lynch et al. (2019) | Long. 2 measures (baseline – follow-up). Dependent variable measured during follow-up (9 months total) | 314, Brazil, (start) 11-17 yrs, (end) 11-18 yrs organized sport | Investigate the effects of different sports on the incidence of traumatic fractures (TF; sport-related fractures and those occurring in daily activities) among adolescents during the 9-month follow-up period | Child.,  Adol., no sport | Adolescents engaged in sports showed a lower incidence of traumatic fractures than nonengaged adolescents | Traumatic fractures |
| Mattila et al. (2008) | Long. Baseline data were collected for each cohort between 1979 to 1997 and followed for an average of 11 years. Follow- up measure in year 2001, date of first lower back pain hospitalization or date of death. | 57408, Finland, (start) 14, 16, 18 yrs,(end) 14-41 yrs, organized and non-organized sport | Investigate whether health, physical activity and other health behaviors, socio-demographic background, and school success predict LBP hospitalization until early middle age | Adol., other physical exercise | Among females, participation in organized sports was associated with an increased risk for low back pain hospitalization persisting into adulthood | Hospitalization |
| *Mattila et al. (2009) | Prospective cohort study. Baseline data were collected for each cohort between 1979 to 1997 and followed for an average of 10.6 years. Follow-up time range from 0-23 years. | 57408, Finland,  14-18 yrs (start), 14-41 yrs (end), organized and non-organized sport | Investigate the nature and risk factors of injuries leading to hospitalization | Adol., other leisure time physical activity | Health behaviour in adolescence, particularly sports club activity, predicted injury hospitalization | Hospitalization |
| *McVeigh et al. (2019) | Long. 5 measures of sport participation (ages: 5, 8, 10, 14, 17) and bone mass at age 20 (15 years total) | 984, Australia, 5 yrs (start), 20 yrs (end), organized sport | Investigate the relationship between organized sports trajectories and bone mass at age 20 years | Child., parent, no sport | Participation in organized sport during childhood and adolescence is associated with bone mass at age 20 years | Bone Mass |
| *Moeijes et al. (2018) | Long. 2 measures (grade 4, 5; 1 year total) | 695, Netherlands, 4^th^ grade (start), 5^th^ grade (end), organized sport | Explore longitudinal associations between several characteristics of sports participation and three aspects of psychosocial health (internalizing problems, externalizing problems, and pro-social behaviour) in Dutch children aged 10– 12 years | Child., no sport | Sport club participation was associated with fewer internalizing problems and better pro-social  behaviour. Fewer internalizing problems were also associated with the kind of sports participation. | Internalizing problems  Externalizing problems  Pro-social behaviors |
| *Moeijes et al. (2019) | Long. 2 measures (grade 4, 5; 1 year total) | 618, Netherlands, 4^th^ grade (start), 5^th^ grade (end), organized sport | Explore longitudinal associations between sports participation and health-related quality of life (HRQoL.) | Child., no sport | Children should be encouraged to perform any kind of sports activity on a very regular base | HRQoL |
| *Murray et al. (2021) | Long. 21 survey cycles (grade 7 to 11; 8 years total) | 1294, Canada, 7^th^ grade (start), 3 years post-high school (end), organized team sport | Study the association between pattern of team sport participation from adolescence to young adulthood and mental health in young adulthood. | Adol., no sport | Individuals who sustained in sport from adolescence to young adulthood reported lower stress and better coping levels compared to non-participants and dropouts. | Anxiety, depressive symptoms, stress |
| *Palomäki et al. (2018) | Long. measures assessed for each variable (ages: 3, 6, 9, 12, 15, 18, 37, 40, 43; 28 years total) | 1285, Finland, 9-18 yrs (start), 37-43 yrs (end), organized sport | Investigate the association between participation in organized youth sport and adult healthy lifestyle habits | Child., adol. no sport | Youth sport participants were more likely to have healthy habits in adulthood than non-sport participants | Physical activity |
| *Perron et al. (2012) | Long. measures assessed for each variable (ages: 7, 8, 10; 3 years total) | 1250, Canada, 7 yrs (start), 10 yrs (end), organized sport | Test whether sports participation moderates the negative effect of peer victimization on elementary school-aged children’s internalizing and externalizing mental health | Child., parent no sport | Sports participation may be beneficial regarding several aspects of development, specifically for children who suffer from peer victimization. Benefits vary depending on type of sports played. | Depressive symptoms  Externalizing problems  Peer victimization |
| *Pfeiffer et al. (2006) | Long. 3 measures/ time points (grade 8, 9, 12; 4 years total) | 429, USA, 8^th^ grade (start), 12^th^ grade (end), organized team sport | Determine the odds of engaging in future moderate-to-vigorous physical activity (MVPA) and vigorous physical activity (VPA) in adolescent female sport participants | Adol., no sport | Adolescent girls who participate in sports in eighth, ninth, and twelfth grades are more likely to be vigorously active over time. | Physical activity |
| *Sabiston et al. (2016) | Long. 20 survey cycles for sport participation under 5 years. 1 measure for depressive symptoms 3 years after secondary school (cycle 21; 8 years total) | 860, Canada, Secondary school (start), 3 yrs after secondary school (end), organized sport | Examine the longitudinal and unique association between number of years  of team sport and individual sport participation during adolescence and depressive symptoms during early  adulthood | Adol., no sport | Team sport participation may protect against depressive symptoms in early adulthood | Depressive symptoms |
| *Shull et al. (2020) | Long. 2 measures/ time points (grade 7, 9; 2 years total) | 306, USA, 7^th^ grade (start), 9^th^ grade (end), organized sport | Examine associations between sport participation, and objectively assessed physical activity and sedentary behavior in youth during the transition from middle school to high school | Child., no sport | Children’s participation in sport is associated with greater levels of physical activity and lower levels of sedentary behaviour during the transition from middle school to high school | Physical activity  Sedentary behaviour |
| Spiegleret al. (2020) | Long. Cross-sectional. 5 measures/time points for BMI (age: 3, 5, 7, 11, 14). 3 measures/ time points for FFMI/ FMI (age: 7, 11, 14). 3 measures/ time points for club sport participation (age: 5, 7, 11; 11 years total) | 18818, United Kingdom, 3 yrs (start), 14 yrs (end), organized sport | Assess the association of gestational age groups and club sport participation in childhood on body mass index (BMI), fat free mass index (FFMI) and fat mass index (FMI) | Child., parent, no sport | Club sport should be encouraged in both, term and preterm born children | Physical activity  Overweight  Adiposity |
| *Tammelin et al. (2003) | Long. 2 measures/ time points (at start and end of study; 17 years total) | 7794, Finland, 14 yrs (start), 31 yrs (end), organized sport | Evaluate how physical  activity and social status in adolescence are associated with physical inactivity in adulthood | Adol., no sport | Infrequent participation in sports and a low grade in school sports in adolescence was associated with physical inactivity in adulthood | Physical activity |
| Telford et al. (2016) | Long. 6 measures/ time points (age: 8, 9, 10, 11, 12, 16; 8 years total) | 289, Australia, 8 yrs (start), 16 yrs (end), organized sport | Investigate the longitudinal effect of sport participation in physical activity, fitness and body fat changes during childhood and adolescence | Child., adol., no sport | Sports club participants were more active (diminish during adolescence), fitter (over time) and, more physically fit, and had less body fat (girls only) than non-sport participants | Physical activity  Physical fitness Adiposity |
| *Vella & Cliff (2018) | Long. 2 measures/ time points (wave 5 & 6; 2 years total) | 4033, Australia, 12 yrs (start), 14 yrs (end), organized sport | Investigate bidirectional associations between sport-participation and adiposity | Child., parent, no sport | No associations were found in either direction | Adiposity |
| *Vella et al. (2014) | Long. 2 measures/ time points (wave 3 & 4; 2 years total) | 4042, Australia, 8 yrs (start), 10 yrs (end), organized sport | Investigate longitudinal associations between sports participation and HRQoL. in children | Child., parent, no sport | Children who participated in sports had greater HRQoL compared to non-participants and dropouts. Team or team & individual sports had higher HRQoL than individual sport participants | HRQoL. |
| *Vella et al. (2017) | Long. 2 measures/ time points (wave 5 & 6; 2 years total) | 4023, Australia, 12 yrs (start), 14 yrs (end), organized sport | Investigate bidirectional associations between sport participation and mental health during adolescence | Child., parent, no sport | The findings show bidirectional associations between sport participation and mental health in adolescents. | Mental health  Internalizing problems  Externalizing problems |
| Vella et al. (2019) | Long. 3 measures/ time points (wave 4, 5 & 6; 4 years total) | 4286, Australia, 10 yrs (start), 14 yrs (end), organized sport | Explore multiple pathways through sports participation during childhood and adolescence associated with adiposity | Child., parent, no sport | There were no associations (direct or indirect) between sport participation and adiposity | Adiposity |
| *Wagnsson et al. (2014) | Long. 3 measures/ time points, from 3 different cohorts, each year (3 years total) | 1358, Sweden, 10, 13, 16 yrs (start), 12, 15, 18 (end), organized sport | Explore the associations between sport participation and self- esteem across adolescence | Child., adol., no sport | Sport participation is associated with self-esteem mediated by perceived sport competence | Self-esteem  Perceived sport competence |
| *Walters et al. (2009) | Long. 2 measures/ time points (at start and end of study; 5 years total) | 1709, USA, 15-16 yrs (start), 20-21 yrs (end), organized sport | Examine the associations between socioeconomic status, gender, sports participation and MVPA | Adol., no sport | Adolescents showed a decline in MVPA over time. Athletes differed in MVPA over time compared to non-participants. Socioeconomic status had a small moderating effect over time for boys | Physical activity |
| *Wang et al. (2017) | Long. Measured from wave 2 – 7 in three different cohorts (the combined cross sequential sample provided information on participants from grade 1 to grade 12) | 1065, USA, Grade 1^st^, 2^nd^, 4^th^ (start), 9-12^th^ Grade (end), organized sport | Investigate motivational trajectories across childhood / adolescence associated to sport participation and examine the association between sport participation and depressive symptoms | Child., no sport | Team based sport participation predicted decline in depressive symptoms across adolescence | Depressive symptom |
| *Wichstrøm et al. (2013) | Long. 4 measures/ time points (13 years total) | 3251, Norway, 12-19 yrs (start), 25-32 yrs (end), organized sport | Investigate predictors of change in leisure time physical activity from adolescence to young adulthood | Child., adol., no sport | Sports club participation and positive athletic self-concept predict leisure time physical activity later in life | Physical activity |
| *Yang et al. (2009) | Long. 6 measure/time points (baseline, 3, 6, 9, 12, 21 years later)  in six different cohorts (21 years total) | 1493, Finland,  3, 6, 9, 12, 15, 18 (start), 24, 27, 30, 33, 36, 39 (end), organized sport | Investigate the effect of organized sport in childhood/adolescence on metabolic syndrome in adulthood | Child., adol., no sport | Participation in organized sports over 3 years in youth is associated with reduced risk for developing metabolic syndrome in adulthood | Metabolic syndrome |
| *Yang et al. (2010) | Long. 4 measure/time points (baseline, 3, 21, 27 years later) in four different cohorts (27 years total) | 664, Finland,  9, 12, 15, 18 yrs (start), 36, 39, 42, 45 yrs (end), organized sport | Explore the long-term effects of youth leisure time physical activity and sports participation on chronic work stress in adulthood | Child., adol., no sport, not physically active | Leisure time physical activity and sports participation in childhood/adolescence predicted lower chronic work strain in adulthood. | Chronic work strain |
| *Zarret & Bell. (2014) | Long. measured from wave 1, 3, 4 (4 years total) | 1482, USA, 7^th^ grade (start), 11^th^ grade (end), organized sport | Examine the effect of out of school time activities on youth weight status through adolescence | Adol., no sport | Participation in sport-dominant activities for 2 or more years had lower odds for at risk overweight/ obesity among adolescents | Overweight/Obesity |

Long. (Longitudinal) ***Adol. (Adolescent): Child. (Children) **** yrs (years) ****** HRQoL. (Health related quality of life)
