# Supplementary material for: Health-related outcomes of youth sport participation: a systematic review and meta-analysis
Source: Int J Behav Nutr Phys Act. 2025 Jul 1;22:89. doi: 10.1186/s12966-025-01792-x (PMC12220085; doi:10.1186/s12966-025-01792-x)
Supplement: Supplementary file 2 — Supplementary Material 2. [file 12966_2025_1792_MOESM2_ESM.docx]

**Bibliography**

References marked with an asterisk (*) signify studies included in the review.

1. *Bedard C, Hanna S, Cairney J. A longitudinal study of sport participation and perceived social competence in youth. J. Adolesc. Health. 2020;66(3):352-359. <https://doi.org/10.1016/j.jadohealth.2019.09.017>

2. *Brunet J, Sabiston CM, Chaiton M, Barnett TA, O'Loughlin E, Low NC, et al. The association between past and current physical activity and depressive symptoms in young adults: a 10-year prospective study. Ann. Epidemiol. 2013;23(1):25-30. <https://doi.org/10.1016/j.annepidem.2012.10.006>

3. *Deshpande SK, Hasegawa RB, Rabinowitz AR, Whyte J, Roan CL, Tabatabaei A, et al. Association of playing high school football with cognition and mental health later in life. JAMA Neurol. 2017;74(8):909-918. <https://doi.org/10.1001/jamaneurol.2017.1317>

4. Dodge T, Lambert SF. (2009). Positive self-beliefs as a mediator of the relationship between adolescents’ sports participation and health in young adulthood. J. Youth Adolesc. 2009;38:813-825. <https://doi.org/10.1007/s10964-008-9371-y>

5. Doré I, Sabiston CM, Sylvestre MP, Brunet J, O'Loughlin J, Abi Nader P, et al. Years participating in sports during childhood predicts mental health in adolescence: A 5-year longitudinal study. J. Adolesc. Health. 2019;64(6):790-796. <https://doi.org/10.1016/j.jadohealth.2018.11.024>

6. Fay K, Lerner RM. Weighing in on the issue: A longitudinal analysis of the
influence of selected individual factors and the sports context on the developmental trajectories of eating pathology among adolescents. J. Youth Adolesc. 2013;42:33-51. <https://doi.org/10.1007/s10964-012-9844-x>

7. Golle, K., Granacher, U., Hoffmann, M., Wick, D., & Muehlbauer, T. (2014). Effect of living area and sports club participation on physical fitness in children: a 4-year longitudinal study. *BMC Public Health*, *14*, 1-8.

8. Mattila VM, Saarni L, Parkkari J, Koivusilta L, Rimpelä A. Predictors of low back pain hospitalization–a prospective follow-up of 57,408 adolescents. Pain. 2008;139(1):209-217. <https://doi.org/10.1016/j.pain.2008.03.028>

9. *Moeijes J, van Busschbach JT, Bosscher RJ, Twisk JW. Sports participation and health-related quality of life: a longitudinal observational study in children. Qual. Life Res. 2019;28:2453-2469. <https://doi.org/10.1186/s12955-019-1124-y>

10. Spiegler J, Mendonça M, Wolke D. Association of sport participation in preterm and full term born children and body and fat mass indices from age 3 to 14 years. JSAMS. 2020;23(5):493-7. <https://doi.org/10.1016/j.jsams.2019.11.006>

11. Telford RM, Telford RD, Cochrane T, Cunningham RB, Olive LS, Davey R. The influence of sport club participation on physical activity, fitness and body fat during childhood and adolescence: The LOOK Longitudinal Study. JSAMS. 2016;19(5):400-6. <https://doi.org/10.1016/j.jsams.2015.04.008>

12. Wichstrøm L, von Soest T, Kvalem IL. Predictors of growth and decline in leisure time physical activity from adolescence to adulthood. Health psychol. 2013;32(7):775. <https://doi.org/10.1037/a0029465>

13. *Yang X, Telama R, Hirvensalo M, Hintsanen M, Hintsa T, Pulkki-Råback L, Mansikkaniemi K, Viikari JS, Keltikangas-Järvinen L, Raitakari OT. Sustained involvement in youth sports activities predicts reduced chronic job strain in early midlife. J. Occup. Environ. Med. 2010;52(12):1154-9. <https://doi.org/10.1097/JOM.0b013e3181fe68bf>

14. Zarrett N, Bell BA. The effects of out-of-school time on changes in youth risk of obesity across the adolescent years. J. Adolesc. 2014;37(1):85-96. <https://doi.org/10.1016/j.adolescence.2013.11.001>
