# Supplementary figures and images for: Health-related outcomes of youth sport participation: a systematic review and meta-analysis
Source: Int J Behav Nutr Phys Act. 2025 Jul 1;22:89. doi: 10.1186/s12966-025-01792-x (PMC12220085; doi:10.1186/s12966-025-01792-x)

**Additional file 3**

*Funnel plot of potential publication bias*


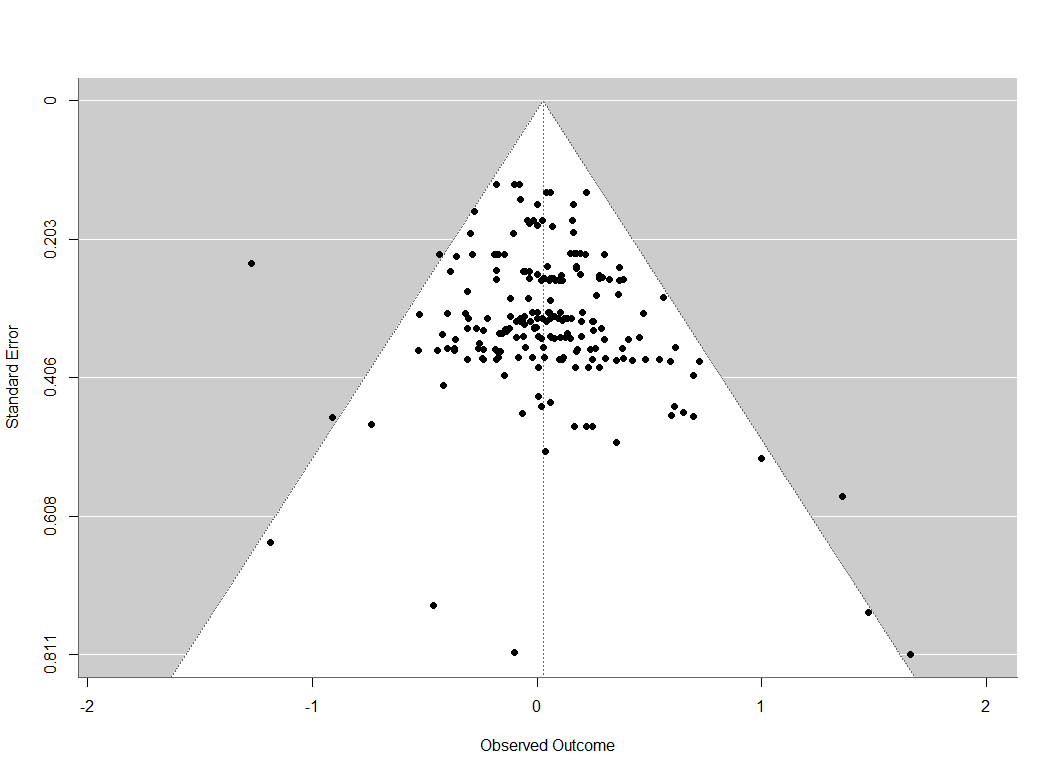

Supplement: Supplementary file 4 — Supplementary Material 4. [file 12966_2025_1792_MOESM4_ESM.docx]
